# Electronic Health Record Data Can be Used at the Bedside to Identify Older Hospitalized Patients With Delirium

**DOI:** 10.1093/geroni/igaa057.447

**Published:** 2020-12-16

**Authors:** Ariba Khan, Kayla Heslin, Michelle Simpson, Michael Malone

**Affiliations:** 1 Advocate Aurora Health Care, Milwaukee, Wisconsin, United States; 2 Aurora Research Institute, Milwaukee, United States; 3 Advocate Aurora Health Care, Milwaukee, United States; 4 Advocate Aurora Health, Milwaukee, Wisconsin, United States

## Abstract

Delirium is a serious condition that is often underrecognized. Several delirium predictive rules can assist in early detection. The coupling of prediction rules with features of the EHR are in their infancy but hold potential. This study aimed to determine variables within the EHR that can be used to identify older hospitalized patients with delirium. This is a prospective study among patients >=65 years admitted to the hospital. Researchers screened daily for delirium using the 3-D CAM. Predictive variables were extracted from the EHR. Basic descriptive statistics were conducted. Chi-squared and Fischer’s exact tests were used to compare differences among those diagnosed with or without delirium as appropriate; binary logistic regression was used for multivariate modeling. Among 408 participants, mean age was 75 years, 61% were female, and 83% were black. The overall rate of delirium was 16.7% (prevalent delirium 10.5%; incident delirium 6.1%). There was no statistical difference in 30-day mortality (2.9% vs. 2.7%) or 30-day readmission (13.2% vs. 14.7%) rates between those with and without delirium (both P>0.05). Even so, patients with delirium were older, more likely to have a diagnosis of infection and/or cognitive impairment, as well as increased severity of illness (all P’s <0.05). Moreover, patients with delirium had a lower Braden score and higher Morse fall score (both P’s <0.01). In multivariate analysis, cognitive impairment (OR 5.49; 95% CI 2.77-10.87) and lower Braden scores (OR 1.29; 95% CI 1.18-1.41) remained significant predictors of delirium. Further research is needed to develop an automated EHR prediction model.

